# Polycystic intrahepatic infection caused by *Enterococcus casseliflavus*: a case report and literature review

**DOI:** 10.1186/s12882-024-03531-z

**Published:** 2024-03-06

**Authors:** Senyin Xu, Bin Huang, Youjun Cao, Zhongyong Zhong, Jiazhen Yin

**Affiliations:** 1 Department of Ultrasound, Zhejiang Hospital, Hangzhou, Zhejiang China; 2 Department of Diagnostic Radiology, Hangzhou TCM Hospital Affiliated to Zhejiang Chinese Medical University, Hangzhou, Zhejiang China; 3 Department of Nephrology (Key Laboratory of Management of Kidney Disease in Zhejiang Province), Hangzhou TCM Hospital Affiliated to Zhejiang Chinese Medical University, No. 453, Tiyuchang Road, 310009 Hangzhou, Zhejiang China

**Keywords:** *Enterococcus casseliflavus*, Cyst infection, Polycystic liver disease, Percutaneous puncture

## Abstract

**Background:**

*Enterococcus casseliflavus* is a rare pathogenic bacterium that is characterized by vancomycin resistance and can lead to multiple infections in the human body. This report describes a rare case of polycystic intrahepatic infection with *E. casseliflavus* which necessitated antibiotic treatment and surgical intervention involving cystic drainage.

**Case Presentation:**

A 59-year-old woman, a long-term hemodialysis patient, was hospitalized due to a 5-day history of fever, abdominal pain, and diarrhea, which were possibly caused by the ingestion of contaminated food. Her blood culture yielded a positive result for E. casseliflavus, and she was initially treated with piperacillin/tazobactam and linezolid. Later, the antibiotic regimen was adjusted to include meropenem and linezolid. Despite treatment, her body temperature remained elevated. However, subsequent blood cultures were negative for E.casseliflavus.Conventional CT scans and ultrasound examinations did not identify the source of infection. However, a PET-CT examination indicated an intrahepatic cyst infection. Following MRI and ultrasound localization, percutaneous intrahepatic puncture and drainage were performed on the 20th day. Fluoroquinolones were administered for 48 days. On the 32nd day, MRI revealed a separation within the infected cyst, leading to a repeat percutaneous drainage at a different site. Subsequently, the patient’s temperature returned to normal. The infection was considered resolved, and she was discharged on the 62nd day. Follow-up results have been favorable thus far.

**Conclusions:**

Based on the findings from this case, it is recommended to promptly conduct PET-CT examination to exclude the possibility of intracystic infection in cases of polycystic liver infection that are challenging to control. Furthermore, timely consideration should be given to puncture drainage in difficult cases.

## Background

Polycystic kidney disease often involves various organs of the body, and polycystic liver is the most common extrarenal manifestation [1]. In fact, 80% of patients with polycystic kidney disease have polycystic liver, but only 5% of those with polycystic liver have clinical manifestations 2. Infection of the polycystic liver can occur as a result of blood dissemination via the portal vein, and the main manifestation of cyst infection in polycystic liver is fever [3, 4]. Usually, intracystic infection of a polycystic liver is difficult to treat, and it does not resolve spontaneously. Because the infection occurs in a closed cavity and the site is deep, it is difficult to completely control the intracystic infection with conventional anti-infection treatment [3].

This article describes a case of polycystic hepatic cyst infection caused by the rare pathogen *Enterococcus casseliflavus* that was treated with a combination of anti-infection regimens and cyst drainage and had a good outcome.

## Case report

A 59-year-old patient, who has been undergoing hemodialysis for six years due to polycystic kidneys, was transferred from a local hospital to our medical facility due to symptoms of fever and diarrhea.The patient’s mother, two brothers, two sisters, and daughter all have polycystic kidney disease. Five days before admission, the patient experienced abdominal distension and pain, likely caused by consuming spoiled peaches. Over the 5-day period, she had watery stools more than 10 times a day. During her regular hemodialysis session, she developed chills and a high fever (body temperature of 39 °C). An anti-infection treatment regimen consisting of ceftriaxone, imipenem, and cilastatin was initiated. After treatment, the patient’s diarrhea symptoms improved, but her fever persisted.A blood culture test at the local hospital revealed that she was positive for E. casseliflavus, with Enterococcus spp. isolated from two culture vials. The susceptibility profile included: vancomycin’resistant (MIC: 4 µg/mL), ampicillin’sensitive, daptomycin’sensitive, dalfopristin/quinopristin’resistant, gentamicin synergy’sensitive, ciprofloxacin’sensitive, and linezolid’sensitive.

On admission, elevated procalcitonin (16.28 ng/ml), low WBC (2.79 × 10^9^/L with 83.3% neutrophils), anemia (hemoglobin 73 g/dL), thrombocytopenia (platelets 94 × 10^9^/L), high tumor markers (CA 125 at 110.50 U/ml, CA 199 at 1579.24 U/ml), and increased CRP (216.9 mg/L) indicated a severe infection, leading to the start of piperacillin/tazobactam and linezolid.

**Fig. 1:**
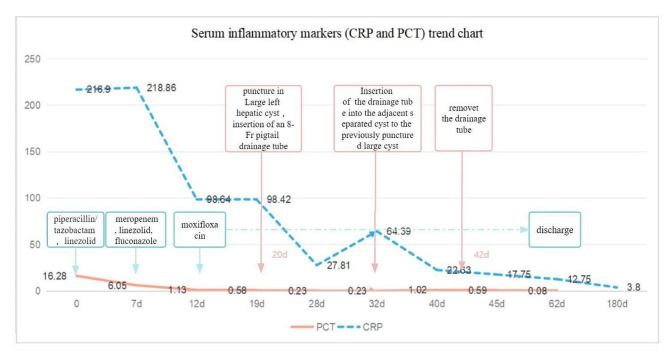
Images of the intrahepatic cysts. a: Full abdominal CT with enhanced arterial phase shows no enhancement of infected cysts. b: FDG metabolism in the cyst wall of the larger cyst of the left liver was slightly increased. c: MRI examination of the T2-stage cross-section showed that the size of the left liver cyst was about 8.9 × 8.8 × 9.0 cm. T2 lipo-pressurization sequence showed that the lesion had a band-like high-intensity shadow, and the cyst wall was slightly thickened. d: MRI examination of the T2 sagittal plane. There was a separation within the cyst, as indicated by the arrow

**Fig. 2:**
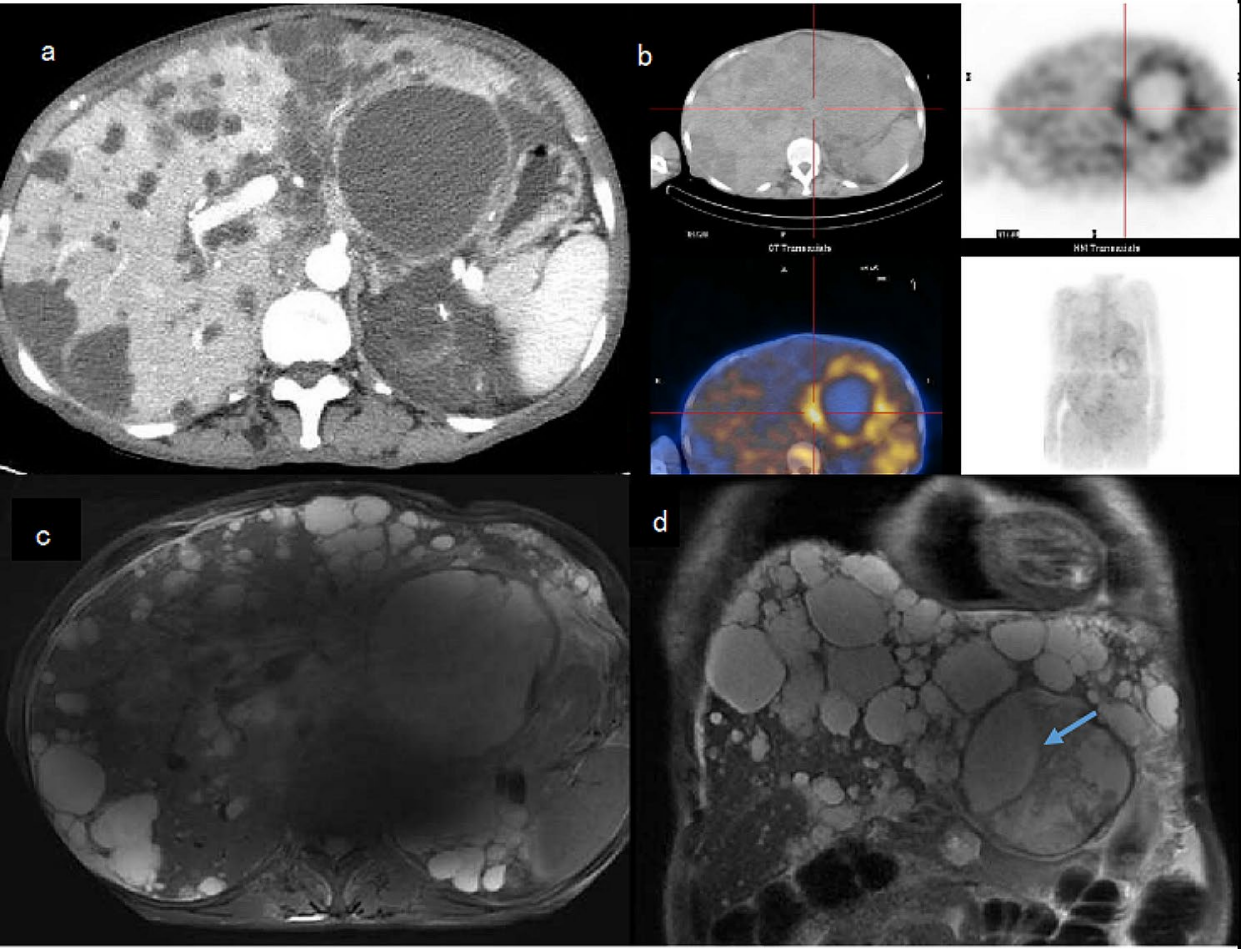
Serum inflammatory markers (CRP and PCT) trend chart

On the 7th day of hospitalization, the patient’s temperature rose to 39.0 °C again. An enhanced CT scan of the abdomen revealed polycystic kidney, polycystic liver, and abdominal pelvic effusion (see Fig. 1a). A revised anti-infection treatment regimen, including meropenem, linezolid, and fluconazole, was administered.The patient’s daily maximum body temperature ranged between 37.3 and 38.5 °C, and she reported abdominal pain. On the 12th day of hospitalization, the patient still experienced fever and abdominal pain. Laboratory tests were conducted again, revealing elevated inflammation markers (see Fig. 2 for details). The patient was intravenously administered moxifloxacin for 49 days until discharge, and PET-CT imaging showed increased fluorodeoxyglucose (FDG) metabolism in the cyst wall of the large left liver cyst (Fig. 1b). A plain upper abdominal MRI scan revealed polycystic liver, polycystic kidney, Some high-signal areas are visible within the liver cysts (Fig. 1c and d), which may be indicative of bleeding.

**Fig. 3:**
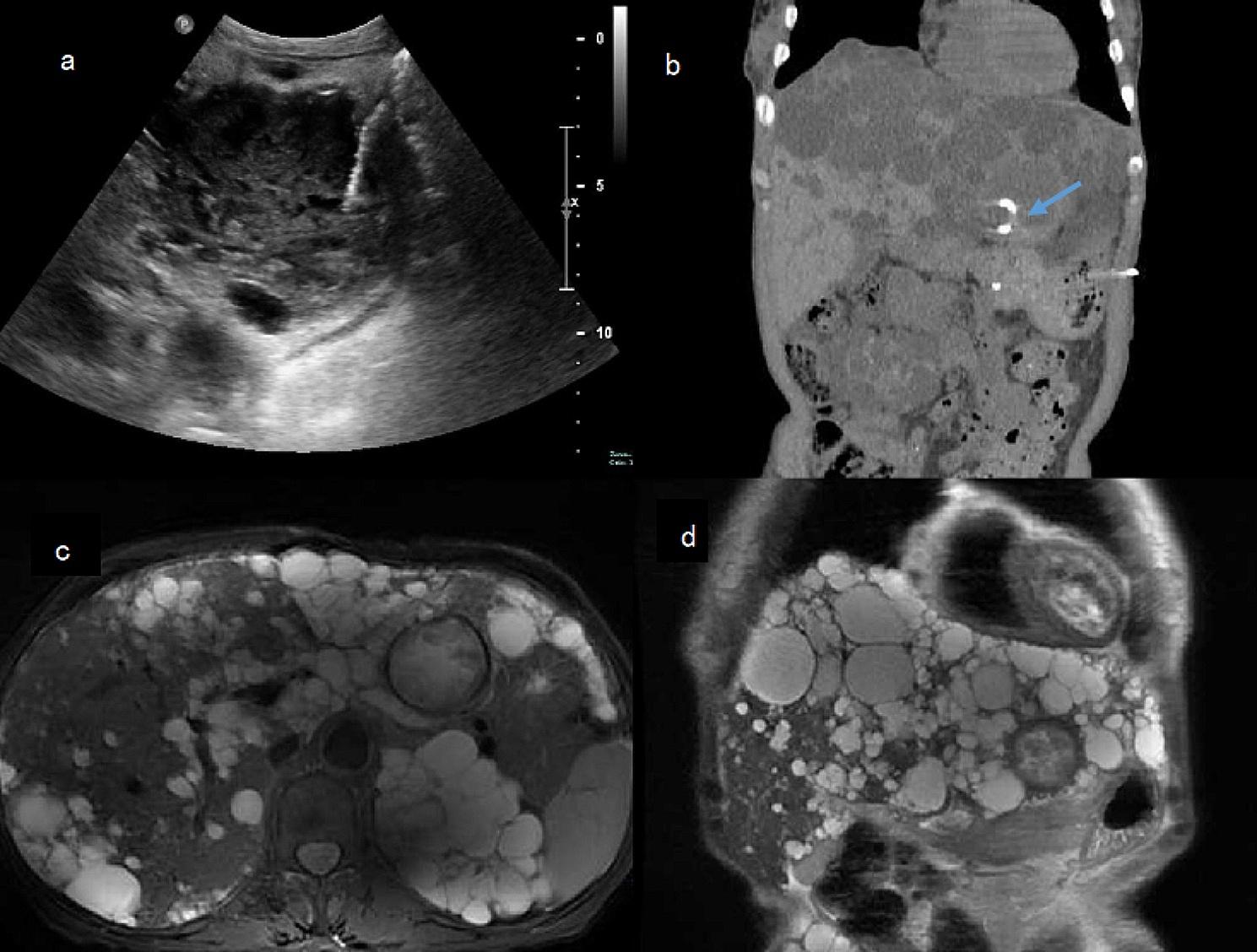
Puncture and insertion of the drainage tube. a: Ultrasound-guided puncture of the infected liver cyst was performed, and an 8-Fr pigtail drainage tube was successfully inserted. b: The drainage tube shadow (arrow) is seen in the cystic lesion of the left lobe of the liver, and the lesion was smaller than before. c: Puncture MRI examination of the T2 cross-section after drainage. d: MRI examination of the T2 sagittal plane after puncture drainage

On the 20th day of hospitalization, an 8-Fr pigtail drainage tube was successfully inserted by ultrasound-guided puncture of the large left hepatic cyst (Fig. 3a), and 160 ml of dark brown liquid was drawn out. The isolate from Cyst fluid culture bottles was identified as *E. casseliflavus*. The colonies in standard Columbia agar exhibited distinct yellow pigment, the coccus was positive for motility. The drainage tube contained a dark brown liquid that flowed out at the rate of 0–50 ml/day, and the body temperature fluctuated between 37.5 and 38.0 °C.

At 32th days after admission, the patient’s body temperature rose again to 38.2 °C.Abdominal MRI revealed cyst separation (Fig. 1c and d). The post-drainage fluid culture yielded the same results as before, continuing treatment with Moxifloxacin hydrochloride.

On the 47th day of admission, no significant fluid collection was observed at the drainage site. The drainage tube was removed, and re-examination showed improved inflammation markers (Fig. 2). During follow-up, CT and MRI revealed a reduced left hepatic cyst with limited diffusion of the cyst wall (Fig. 3b, c, d). The patient was discharged on the 62nd day. After discharge, the patient’s body temperature stayed normal, and follow-up results were favorable.

## Discussion and conclusions

The patient in this case report had polycystic kidney disease combined with polycystic liver. When she was referred to our hospital, she had end-stage renal disease for which she was on regular hemodialysis treatment, and her blood culture was positive for *E. casseliflavus*, a rare pathogen. Continuous antibiotic treatment and monitoring indicated that she had a cystic infection in the liver. Five days before her hospitalization, she had intestinal symptoms. Thus, the intestinal bacteria could have entered the bloodstream and then disseminated through blood to result in secondary liver cyst infection.

The diagnosis and management of polycystic liver disease (PLD) cyst infections present significant challenges due to the absence of definitive laboratory test results and the difficulties associated with detecting intracystic infections through imaging studies. In clinical case reports, an elevation in serum CA19-9 levels relative to baseline measurements has been noted. However, CA19-9 is not a specific marker for cyst infection. The presence of fever (temperature > 38.5 °C for more than 3 days) without other identifiable sources of infection, detection of gas within a cyst by CT or MRI, increased FDG uptake in the cyst lining compared to the normal liver parenchyma as seen on 18FDG PET-CT, tenderness in the liver region, elevated C-reactive protein levels, increased white blood cell count (> 11,000/L), or positive blood cultures should prompt consideration of infection [5, 6]. Conventional radiological imaging has limited utility in confirming hepatic cyst infections due to the lack of reliable features that distinguish between infected and non-infected hepatic cysts [7, 8]. Inflammatory parameters, such as enhanced wall thickening, high signal intensity on diffusion-weighted imaging, fluid-fluid levels, or the presence of intracystic gas bubbles observed on CT or MRI, may support the diagnosis of a hepatic cyst infection.Lantinga [9]concluded that although analyzing cyst fluid is the definitive benchmark, employing a mix of clinical signs, biochemical indicators, and 18 F-FDG PET/CT imaging offers an effective diagnosis in situations where obtaining samples is challenging.In this case, PET-CT proved to be a promising method for detecting infection, and MRI helped determine the location of the infected cyst for interventional treatment.

The 2015 CARI guidelines suggested [3] that once multiple cystic liver infection or a suspicious infection is diagnosed, antibiotics should be used first to treat the infection. Because the infection occurs in the capsule, quinolones with strong lipid solubility are preferred. If the symptoms are not relieved within 72 h, third-generation anti-infection treatment with ceftriaxone should be added to the regimen and the antibiotic regimen should be adjusted according to drug sensitivity and culture results. In the 2022 guidelines [10], it is strongly recommended to use Fluoroquinolones and third-generation cephalosporins as first-line antibiotics for treating hepatic cyst infections, based on a level of evidence 2 and a 90% consensus. Additionally, a 4 to 6 weeks course of antibiotic therapy is advised, backed by a level of evidence 4 and a unanimous 100% consensus.Suwabe’s research indicates that in patients experiencing liver cyst infections, especially those with recurrent episodes and liver enlargement, Gram-negative bacteria constitute 74–79% of the identified pathogens, among which E. coli exhibits notable resistance to fluoroquinolone antibiotics. This study further proposes that the heightened resistance to fluoroquinolones could be associated with prolonged and repeated antibiotic usage [11].In patients with Polycystic Liver Disease (PLD) experiencing cyst infections, surgical treatment options include cyst fenestration, also known as deroofing, and segmental hepatic resection, which are applicable for patients with superficial large cysts or small to medium-sized cysts confined to a few liver segments [12, 13]. These procedures can effectively reduce cyst volume and alleviate symptoms but do not alter the natural progression of the disease. However, they are associated with high morbidity, especially in the presence of vascular abnormalities and biliary dilatation, and may lead to various postoperative complications such as ascites, hemorrhage, pleural effusion, with mortality rates exceeding 2% and a high recurrence rate [14, 15]. Therefore, the potential risks and benefits should be carefully weighed when considering these treatment options.In such scenarios, drainage of the infected cysts becomes a viable option, especially for those presenting with sizable cysts exceeding 5 cm in diameter [16, 17]. However, the intricacies of PLD necessitate a cautious approach to drainage procedures. The complexity lies in accurately pinpointing the infected cyst among many, coupled with the inherent risk of propagating the infection to neighboring cysts [6, 18]. Hence, the drainage technique—whether percutaneous or laparoscopic—should mirror the strategies employed for managing pyogenic liver abscesses, ensuring meticulous execution to mitigate potential complications [16, 17]. In this case, the patient initially presented with high fever, elevated inflammatory markers, and signs of sepsis. At the local hospital, cephalosporins and carbapenems (imipenem) were sequentially administered. Upon admission to our hospital, we initiated treatment with ampicillin/sulbactam and linezolid. However, due to persistent fever, the antibiotic regimen was changed to meropenem and linezolid. Considering the poor tissue penetration of meropenem for liver cyst infection [19], moxifloxacin was subsequently prescribed.In the present case, it was difficult to control the infection in the early stage of antibiotic treatment. Therefore, the cyst was punctured and drained under ultrasound guidance. At the first drainage, 160 mL of dark brown liquid was drawn. Following this, the patient’s body temperature reduced. Because of the separation of the cyst observed on an MR scan, cyst puncture and drainage were performed at two sites. Later, because of cyst infection, moxifloxacin was used for anti-infection treatment.

In this case, the blood culture before admission showed a positive result for *E. casseliflavus*, and the culture of the aspirated cyst fluid also confirmed the presence of *E. casseliflavus*. This indicates that the patient had both bacteremia and a liver cyst infection caused by this bacterium. In the United States, vancomycin-resistant Enterococcus has gradually become an important cause of invasive infections [20, 21]. A study evaluating cases of bacteremia caused by *E. casseliflavus* and *E. gallinarum* showed that malignancy (70.0%) and diabetes mellitus (20.0%) were the most common complications in these bacteremia cases, suggesting that *E. casseliflavus* bacteremia is more likely to occur in immunocompromised hosts [16]. During the patient’s hospitalization, tumor markers CA125 and CA199 were elevated, but no solid tumors were identified. Long-term dialysis was also identified as a risk factor for colonization by *E. casseliflavus* [22, 23]. In a survey conducted in the United States, the 30-day all-cause mortality rate of *E. casseliflavus* patients was found to be 10.4%, and the comprehensive treatment failure rate was 39.6% [24].

*E. casseliflavus* carries the VanC gene intrinsically and exhibits moderate resistance to vancomycin (VCM). Due to this moderate resistance, the Clinical and Laboratory Standards Institute (CLSI) considers VCM concentrations below 4 mg/dL to be susceptible for Enterococcus species, and therefore, the drug is not actively recommended for the treatment of *E. casseliflavus* [25, 26] In fact, the minimum inhibitory concentration (MIC) of VCM demonstrated in this case was 4 mg/dL. It is important to note that even if microbiological tests show that this microorganism is susceptible to VCM, the actual therapeutic effect of VCM is likely to be low [25, 26]. However, most strains of E. casseliflavus are sensitive to penicillin and ampicillin. Therefore, combination therapy with ampicillin and aminoglycoside drugs such as gentamicin or streptomycin is considered the standard treatment [27]. Since most *E. casseliflavus* strains are negative for β-lactamase, meropenem does not have a better effect than ampicillin in the treatment of enterococcal infections. Thus, in the reported cases, there is no clear indication for combination therapy with meropenem and β-lactamase inhibitors [28]. In this case, the patient was treated with meropenem and β-lactamase inhibitors, but the infection remained difficult to control. This may be explained by the deep location of the infection site within the hepatic cyst.

The difficulty in treating *E. casseliflavus* infection in polycystic liver disease may be attributed to several factors. Firstly, the lesions in polycystic liver disease are typically located deep within the liver, making it challenging for antibiotics to effectively penetrate the infection site. Secondly, E. casseliflavus exhibits intrinsic resistance to vancomycin under natural conditions, rendering conventional treatment with vancomycin ineffective. Additionally, the multidrug resistance and spread of resistant genes in *E. casseliflavus* contribute to the difficulty in treating the infection. Promising outcomes were achieved after full puncture and drainage in this case. The patient is currently undergoing regular dialysis treatment, and the levels of inflammation markers, albumin, and other nutritional indicators have returned to normal. Therefore, in cases of difficult-to-control polycystic liver infection, immediate PET-CT examination is recommended to exclude the possibility of intracystic infection and promptly perform puncture drainage as an interventional treatment.

## Data Availability

Data sharing is not applicable to this article as no datasets were generated or analyzed.

## Abbreviations

PCT: Procalcitonin
WBC: white blood cell
CRP: C-reactive protein
FDG: fluorodeoxyglucose
